# Endovascular techniques for the treatment of true renal arterial aneurysms—procedural insights and outcomes

**DOI:** 10.1186/s42155-025-00611-5

**Published:** 2025-10-30

**Authors:** Arjan Singh Khattar, Raj Das, Joo-Young Chun, Akos Berczi, Lakshmi Ratnam, Seyed Ameli Renani, Ben Hawthorn, Michael Gonsalves, Robert Morgan

**Affiliations:** 1 Department of Interventional Radiology, St George’s University Hospital NHS Foundation Trust, London, SW17 0QT UK; 2 Department of Interventional Radiology, Heart and Vascular Center, Semmelweis University, Varosmajor Utca 68, Budapest, Hungary

**Keywords:** Renal artery aneurysm, Renal artery, Endovascular procedures, Embolization, Vascular surgical procedures, Angiography, Stents, Coils, Percutaneous therapeutic procedures, Treatment outcome

## Abstract

**Purpose:**

To discuss endovascular techniques and report the outcomes for endovascular treatment of true renal arterial aneurysms (TRAAs).

**Materials and methods:**

A 22-year retrospective analysis of endovascular treatment of TRAAs in our institution. Aneurysm characteristics and endovascular techniques are discussed. Outcome measures were technical and clinical success (need for reintervention), renal parenchymal perfusion loss, impact on renal function, and complications. A 30-day mortality analysis was performed. Impact on renal function was assessed with a two-tailed, paired *t*-test of pre- and post-procedural serum creatinine.

**Results:**

Eighteen endovascular procedures were performed to treat 15 TRAAs in 14 patients (including three reinterventions). 14/15 TRAAs were classified as Type 2 and 1/15 as Type 1 (Rundback classification). Mean initial aneurysm size was 22.9 mm (range 5–40 mm).

Of the 14 Type 2 TRAAs, five were initially treated with balloon-assisted Onyx embolisation, four with stent-assisted coiling, four with sac packing, and one with stent-grafting.

The technical success rate was 100%. The TRAAs requiring reintervention had been originally treated with balloon-assisted Onyx embolisation (two TRAAs) and stent-grafting (one TRAA).

Renal parenchymal loss was < 10% in 10/15 TRAAs after initial intervention. At reintervention, 2/3 cases had 60–70% estimated parenchymal loss as the TRAAs had to be treated more aggressively. Complications (grade 1–3) occurred in 5/18 procedures. The grade 2 complication was atrophy and loss of renal function of the treated kidney (with serum creatinine remaining in the normal range) (*n* = 1). Grade 3 complications were brachial access pseudoaneurysm (*n* = 1), common femoral vein thrombosis (*n* = 1), and access site cellulitis (*n* = 1).

**Conclusion:**

Endovascular treatment of TRAAs has a high rate of technical success and a low impact on renal function. A higher rate of reintervention was observed for TRAAs treated with Onyx embolisation, leading to a shift towards stent-assisted coiling as our preferred technique when anatomically feasible.

## Introduction

True renal artery aneurysms (TRAAs) are rare, with an incidence of 0.01% to 0.09% in general autopsy series, and 0.73% to 0.97% in angiographic series [1–3]. They represent 22–25% of visceral arterial aneurysms [4]. The development of TRAAs is associated with several risk factors, including fibromuscular dysplasia, atherosclerosis, hypertension, and various forms of vasculitis [5, 6]. Like other true arterial aneurysms, TRAAs are characterised by a localised dilatation of the arterial wall involving all three layers. Indications for treatment include aneurysm size, patient symptoms (most commonly haematuria and/or pain), rapid growth, rupture, and females who are pregnant or of childbearing age. The size threshold for treatment has historically been 20 mm in asymptomatic patients, but recent Society of Vascular Surgery (SVS) guidelines now recommend a threshold of 30 mm [7]. The evidence for this change remains controversial and the CIRSE Standard of Practice document still supports endovascular treatment for renal aneurysms > 20 mm, especially when the aneurysms are saccular, distally located, and vascular anatomy is favourable [8].

Endovascular treatment (EVT) has gained favour for its high technical/clinical success rates and minimally invasive nature, which enables a shorter hospital stay in comparison with surgical treatment. The choice of endovascular technique is influenced by the anatomical characteristics of the aneurysm and the operator’s experience. The primary objective is to exclude perfusion of the aneurysm such that its growth and risk of rupture are reduced, all while maintaining renal perfusion wherever possible. Techniques such as stent-grafting and mechanical embolisation (with or without stent or balloon assistance) have been reported in the literature, though outcome data remain limited to case series [5–7, 9].

The objective of this study is to expand on the existing literature by reporting our institution's experience with EVT of TRAAs, with particular focus on the endovascular techniques used, technical and clinical success rates, complications, and impact on renal parenchymal perfusion and renal function. This study builds on our publication of 2016, which reported the treatment of nine TRAA cases over 12 years and 10 months [9]. Since that time, we have gathered further data and refined our techniques, thereby enabling us to provide a more comprehensive analysis.

## Materials and methods

This retrospective study incorporated all patients who underwent EVT for TRAAs from January 2002 to May 2024 at a vascular tertiary referral centre in London, United Kingdom. Cases were identified via a retrospective search of the Radiology Information System, and data were acquired by reviewing relevant procedure reports, medical records, and imaging studies. Cases of treated pseudoaneurysms (e.g., traumatic) or multiple microaneurysms associated with an underlying mass (e.g., angiomyolipoma or oncocytoma) were excluded.

**Fig. 1:**
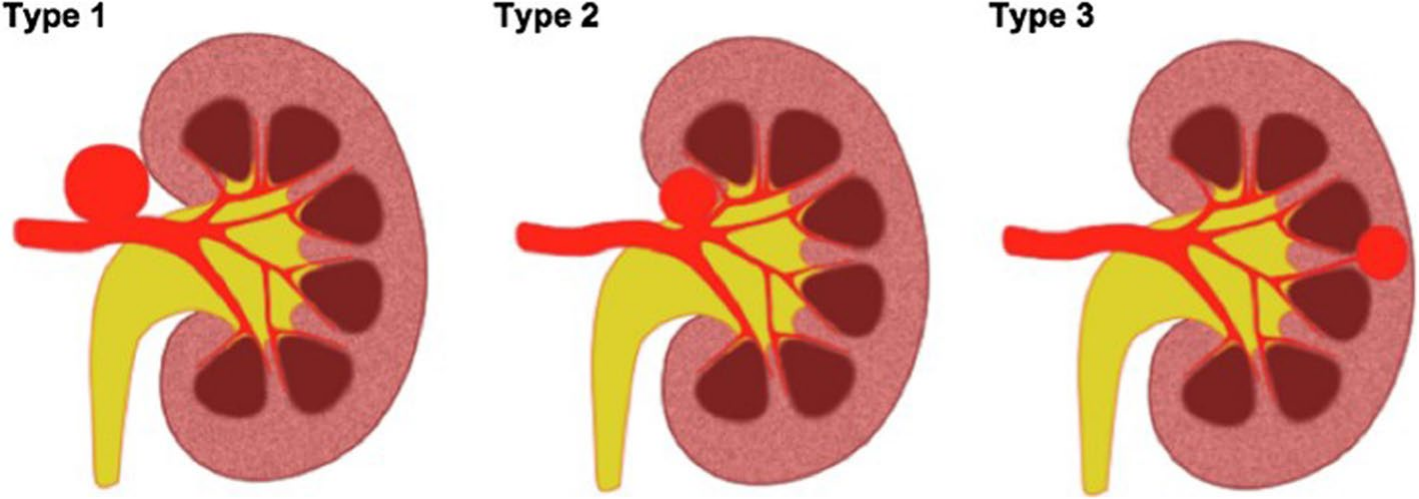
The Rundback Classification of TRAAs. Type 1- arising from main renal artery trunk; Type 2- extrarenal aneurysm arising at the proximal arterial bifurcations; Type 3- aneurysm arising from intrarenal distal arterial branches

Data collected included patient demographics: age, gender, aetiology, indication for treatment, anticoagulant use; aneurysm characteristics: size (mm), side, Rundback classification (Fig. 1; Type 1 involves main renal artery, Type 2 occurs at a proximal renal artery bifurcation, Type 3 involves a distal intrarenal artery) [10], presence of renal artery stenosis or arteriovenous fistula; and endovascular technique, including access site.

Specific ethics committee approval is not required for retrospective studies such as this study at our institution.

### Outcome measures and statistical analysis

The primary outcome measures of this study were technical success, clinical success, estimated renal parenchymal loss, impact on renal function, and complications. Technical success was defined as the exclusion of the aneurysm sac from perfusion on immediate post-treatment angiography. Clinical success was defined as the resolution of symptoms (if initially present) and insignificant reperfusion of the TRAA post-procedure i.e., no requirement for reintervention. Clinical failure and criteria for the need for reintervention were defined by the unsuccessful exclusion of the TRAA sac from perfusion on follow-up imaging and/or recurrent symptoms related to the TRAA.

Renal parenchymal loss was determined following consensus reading by two authors of this study who visually assessed the angiographic images of each intervention. It should be noted that dimercaptosuccinic acid (DMSA) renal nuclear imaging was not routinely performed on patients in this series.

Impact on renal function was assessed by statistically comparing pre- and post-procedural serum creatinine (sCr) for any significant difference by means of a two-tailed, paired *t* test. Post-procedural sCr was measured at approximately 24 h post-procedure for each case. A Shapiro–Wilk test for normality was first performed, which confirmed the normality of the dataset. Note, this was with one outlier excluded as this patient had multiple confounding factors for periprocedural acute kidney injury. This was an Ehler’s Danlos patient with multiple acute medical issues who was admitted to the intensive care unit following cardiac arrest due to acute bleeding and haemodynamic instability secondary to a ruptured hepatic arterial aneurysm with arteriovenous fistula, which was embolised emergently. This patient also had a Rundback Type 2 TRAA with associated distal ischaemia of over half of the kidney. Shortly after the emergent embolisation of the hepatic arterial aneurysm overnight, the TRAA was embolised during working hours.

Complications were classified according to the CIRSE Classification System [11].

### Patient demographics

Following the retrospective search and application of exclusion criteria, 16 TRAAs were identified in 15 patients. One TRAA in one patient had to be further excluded due to lack of imaging and procedural/follow-up documentation. Of the 14 remaining patients, there were 9 females and 5 males (mean age 50.4 years [range 23.3 to 80.1 years]). Aneurysm aetiologies included hypertension (8/15 TRAAs, 53.3%), fibromuscular dysplasia (2/15 TRAAs, 13.3%), and Ehler’s Danlos syndrome (1/15 TRAAs, 6.7%); a clear aetiology was unknown for 4/15 TRAAs (26.7%). An anticoagulant or antiplatelet agent was being taken by 4/14 patients (28.6%) at the time of first intervention. The most common indication for treatment was size (> 20 mm) for 9/15 TRAAs (60%). Other indications for treatment are summarised in Table 1.

**Table 1:** TRAA characteristics and indications for treatment

TRAA Characteristics	*N* = 15 (%)
**Aetiology**	
Hypertension	8 (53.3%)
Fibromuscular dysplasia	2 (13.3%)
Ehler’s Danlos syndrome	1 (6.7%)
Unknown	4 (26.7%)
**Indication for treatment**	
Size > 20 mm	9 (60%)
Risk of enlargement during pregnancy	2 (13.3%)
Ehler’s Danlos with separate visceral aneurysm that ruptured	1 (6.7%)
Other clinical decision/Patient preference	3 (20%)
**Morphology**	
Saccular	14 (93.3%)
Fusiform	1 (6.7%)
**Rundback classification**	
Type 1	1 (6.7%)
Type 2	14 (93.3%)
**Side**	
Right	9 (60%)
Left	6 (40%)
**Concurrent renal artery stenosis**	3 (20%)
**Concurrent arteriovenous fistula**	1 (6.7%)

### Aneurysm characteristics

The morphology of 14/15 TRAAs (93.3%) was saccular; the other remaining TRAA was fusiform (1/15, 6.7%). Mean sac size at the time of first intervention was 22.9 mm (range 5–40 mm). The majority of TRAAs were right-sided (9/15, 60%). One patient had bilateral aneurysms. All but one TRAA (14/15, 93.3%) were located at the region of the main arterial bifurcation, i.e., Type 2 according to the Rundback classification (Fig. 1). One TRAA (6.7%) arose directly from the main arterial trunk, i.e., Type 1. Concurrent renal artery stenosis was present for 3/15 TRAAs (20%). A concurrent arteriovenous fistula was present for 1/15 TRAAs (6.7%), which was adequately treated following embolisation of the aneurysm.

TRAA aetiologies and characteristics have been summarised in Table 1.

### Endovascular technique

All procedures were performed by experienced interventional radiologists under local anaesthesia ± conscious sedation (intravenous fentanyl and/or midazolam). All but one intervention (including reintervention) was performed via femoral access (17/18, 94.4%), the other via brachial access (1/18, 5.6%). Brachial access was selected for this case following an unsuccessful attempt via femoral access due to an acutely downward-angled main renal artery. Radial access was not feasible, as the intended “stent-assisted coiling” technique required sheath placement into the main renal artery and, at that time, radial sheaths of sufficient length were not available.

In our institution’s previous publication of 2016, TRAAs were predominantly treated with a “balloon-assisted Onyx embolisation” (Medtronic, Irvine, CA, USA) technique [9]; however, the range of endovascular techniques used in this up-to-date study is more heterogeneous (Table 2) and also includes techniques such as “stent-assisted coiling”, “sac packing with embolic material”, “stent-graft placement” and “plug placement in proximal main artery”. The majority of TRAAs (5/15, 33.3%) were still initially treated with a “balloon-assisted Onyx embolisation” technique; however no TRAAs were treated with this technique since our previous publication. A significant number of TRAAs were treated with “stent-assisted coiling” and “sac packing with embolic material”, each being used for 4/15 TRAAs (26.7% each).

**Table 2:** Endovascular techniques used for TRAA treatment

Endovascular Technique*	*N* = 15 (%)
Balloon-assisted Onyx embolisation	5 (33.3%)
Stent-assisted coiling	4 (26.7%)
Sac packing with embolic material	4 (26.7%)
Plug placement in proximal main artery	1 (6.7%)
Stent-graft placement	1 (6.7%)

*At first intervention; not including reinterventions

The single case of a fusiform TRAA, arising from the proximal bifurcation, was treated with a “stent-assisted coiling” technique with a bare stent and detachable Ruby (Penumbra, Alameda, CA, USA) coils, due to its morphology.

#### Balloon-assisted onyx embolisation

**Fig. 2:**
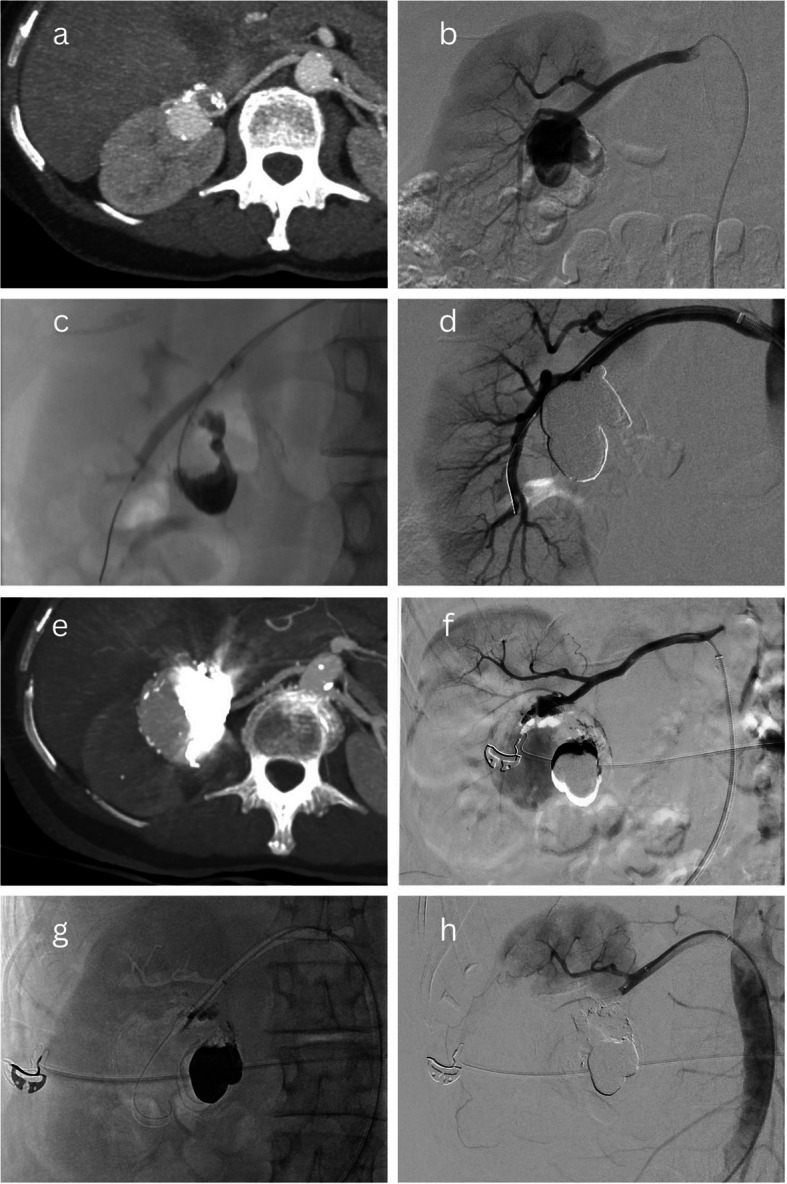
Balloon-assisted Onyx embolisation– Reintervention case 1. A 35-mm saccular Type 2 Rundback aneurysm with two efferent branches (**a**-**b**) was treated endovascularly by catheterising the aneurysm sac and separately manipulating a wire across the aneurysm into an efferent branch and positioning a balloon across the aneurysm’s origin (**c**). The balloon was inflated and the sac was filled with Onyx until successful exclusion (**d**). Four years later, the patient presented acutely with right loin pain and haematuria; CT and angiography demonstrated reperfusion and enlargement of the aneurysm to 56 mm (**e**-**f**). Successful reintervention was performed in an emergent setting by manipulating a wire into the aneurysm sac and embolising the sac and the aneurym’s neck with multiple endovascular occlusion systems and vascular plugs (**g**) at the expense of approximately 66% of renal parenchymal perfusion (**h**). Over time, this kidney became atrophic and non-functioning but biochemical renal function remained within the normal range

“Balloon-assisted Onyx embolisation” describes a technique where the TRAA sac is catheterised and embolised by the injection of Onyx, while a balloon is temporarily inflated across the TRAA neck over a separate wire that has been manipulated across the TRAA into an efferent branch. The aim of this technique is to exclude the TRAA from arterial perfusion while Onyx is injected into the aneurysm sac so that reflux of Onyx into the distal renal arteries is avoided by the inflated balloon. This has been illustrated in Fig. 2a–d.

#### Stent-assisted coiling

**Fig. 3:**
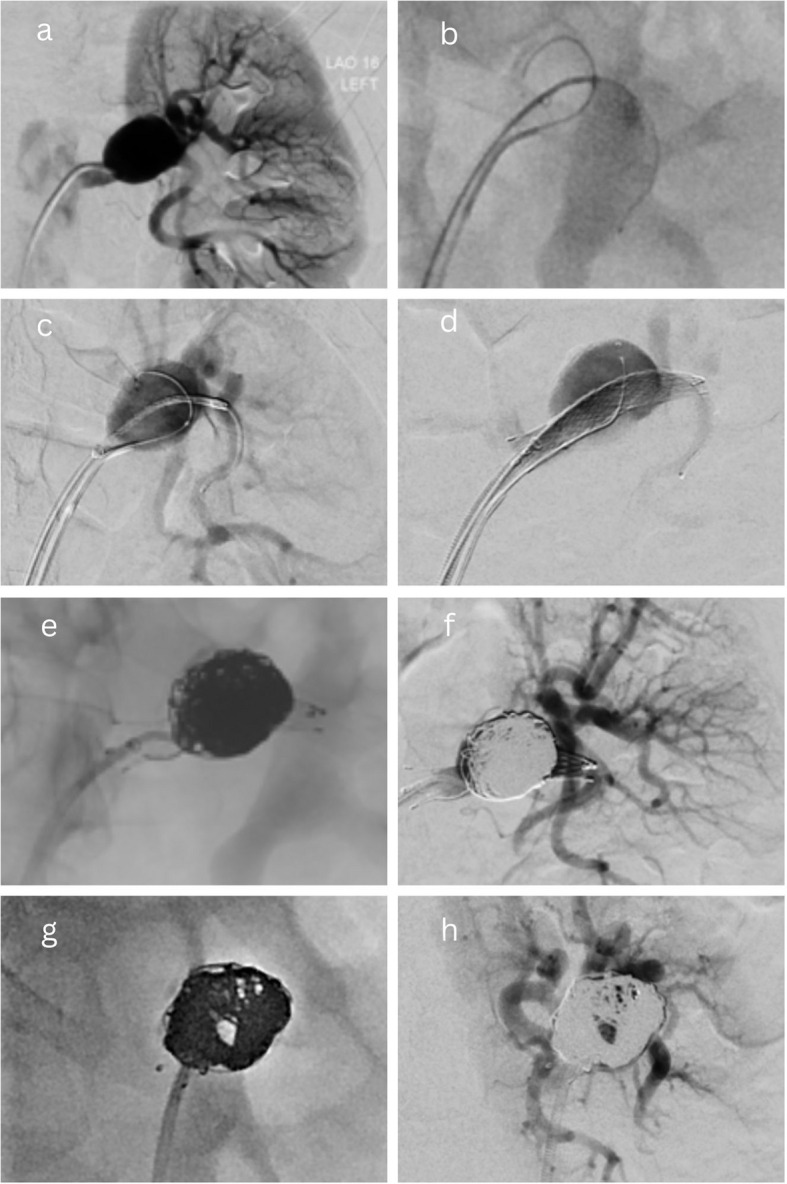
Stent-assisted coiling of a TRAA. A saccular Type 2 Rundback aneurysm (**a**) was treated with stent-assisted coiling. Bilateral femoral arterial access was obtained and both the aneurysm sac and its inferior efferent branch were catheterised (**b**). A self-expanding bare metal stent was deployed across the aneurysm into the inferior efferent branch (**c**-**d**) and multiple coils were deployed into the sac via the trapped microcatheter (**e**), until successful exclusion (**f**). Angiography confirmed a patent channel was maintained through the aneurysm by the stent (“views looking through the stent”) with no loss of renal parenchymal perfusion (**g**-**h**)

“Stent-assisted coiling” was performed using conventional self-expandable bare metal stents; no flow diverter stents (FDS) were used. The stent would be deployed over a wire that had been manipulated across the TRAA into an efferent branch, and then the aneurysm would be embolised with coils via either a microcatheter introduced through the stent mesh or placed within the sac (jailed) before deployment of the stent [12]. An example of this technique has been illustrated in Fig. 3.

#### Sac packing with embolic material

“Sac packing with embolic material” describes a technique where the TRAA sac is catheterised and embolised without the need for balloon or stent assistance. This technique is most suitable when the TRAA is saccular and has a narrow neck with fewer efferent branches, i.e., the risk of inadvertent embolisation of afferent/efferent branches of the TRAA is minimal. The embolic agents utilised for the TRAAs treated with this technique have been summarised in Table 3.

**Table 3:** Embolics used for TRAAs treated with "sac packing" at first intervention

Sac packing with embolic material breakdown	*N* = 4 (%)
Onyx only	1 (25%)
Coils only	1 (25%)
Coils and Onyx	1 (25%)
Occlusion systems, plugs and coils	1 (25%)

#### Plug placement in proximal main artery

**Fig. 4:**
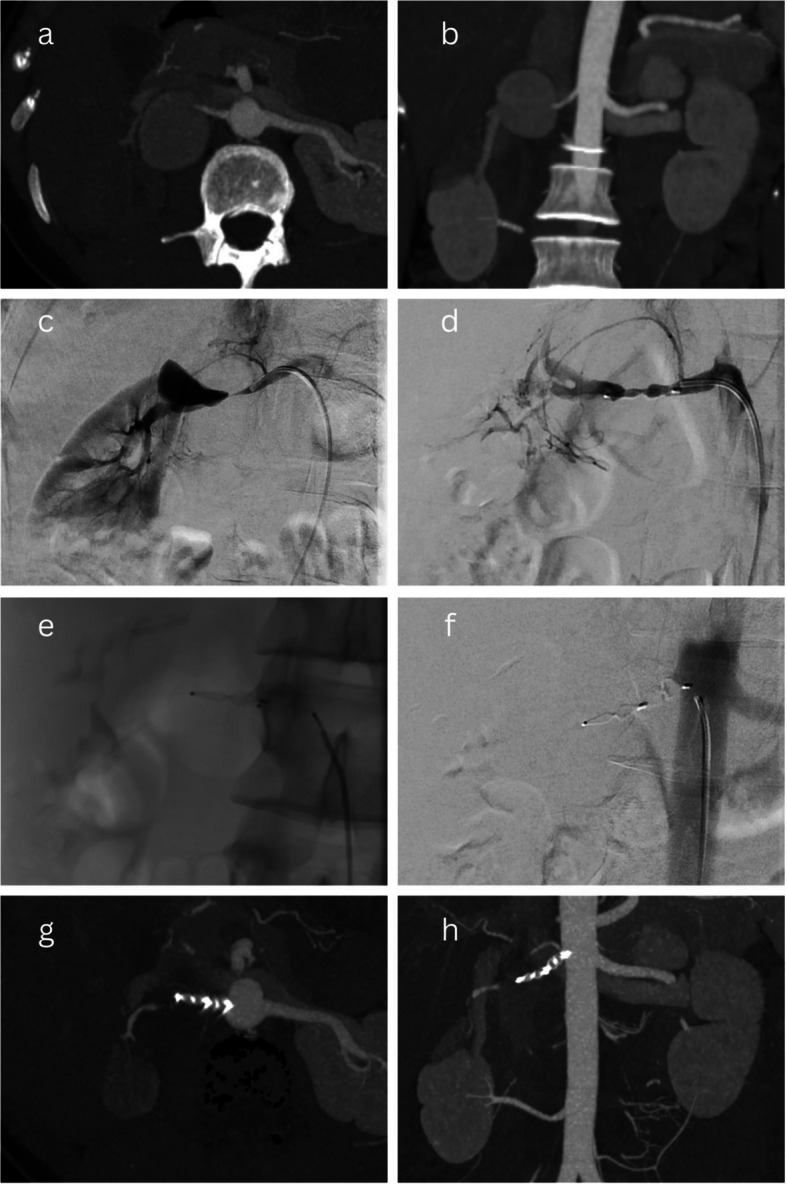
Plug placement in proximal main artery of a TRAA. A saccular Type 1 Rundback aneurysm was treated with plug placement in the proximal main artery supplying the TRAA as it was already associated with distal renal ischaemia (**a**-**b**) and the presence of renal artery stenosis also limited access to the sac (**c**). A wire was manipulated into the sac and a vascular plug was deployed at the proximal TRAA neck (**d**). A further vascular plug was deployed in the proximal main renal artery up to its origin (**e**). Post-treatment angiography confirmed successful exclusion of the aneurysm (**f**). A follow up CT angiogram at 8 weeks confirmed persistent occlusion of the aneurysm (**g**-**h**)

The single case treated with vascular plug (Amplatzer™ Vascular Plug 4, Abbott) placement in the main artery proximal to the TRAA was for the single case of a Type 1 Rundback TRAA. This endovascular technique is not as common for Rundback Type 1 and 2 TRAAs as it makes no effort to preserve any efferent branches of the TRAA. This was deemed appropriate in this case as the TRAA arose from the main upper pole artery and demonstrated renal artery stenosis just proximal to the TRAA, limiting access to the sac. Additionally, the TRAA was already partially thrombosed with established ischaemia of the distal intrarenal branches. This case has been illustrated in Fig. 4.

#### Stent-graft placement

**Fig. 5:**
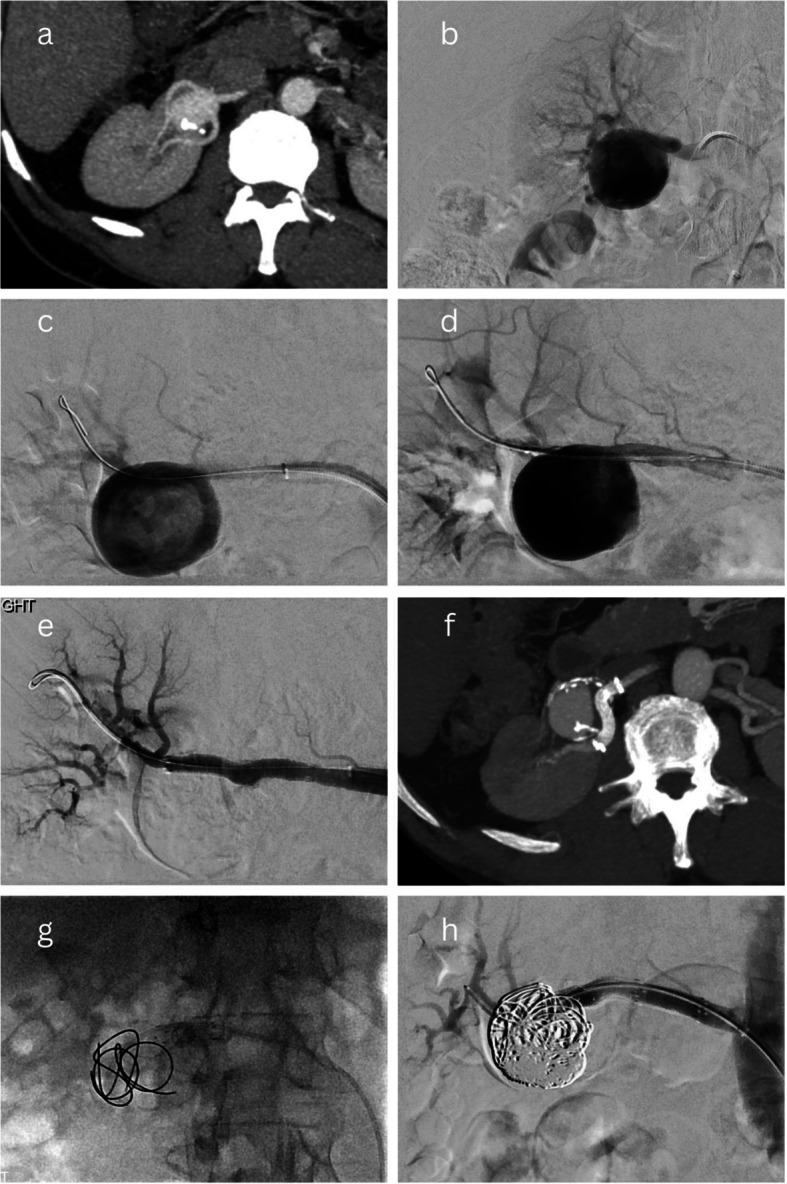
Stent-graft placement— Reintervention case 2. A 37-mm saccular Type 2 Rundback aneurysm with two efferent branches (**a**-**b**) was treated endovascularly by manipulating a wire across the aneurysm, into the posterior division efferent branch (**c**), and placing a covered stent across the aneurysm, successfully excluding it (**d**-**e**). Surveillance CT within the first year demonstrated partial reperfusion of the sac (**f**). At angiography, this was found to be due to an inadequate proximal seal and so a catheter was manipulated alongside the stent and into the aneurysm sac, which was subsequently embolised with coils (**g**), and the covered stent was extended proximally, successful excluding the aneurysm (**h**)

“Stent-graft placement” describes the technique where a covered stent is placed across the neck of the TRAA, over a wire that has been manipulated across the TRAA and into an efferent branch into which the stent-graft is landed. An example of this technique has been illustrated in Fig. 5a–e. Consideration of vascular anatomy is important for this technique, as TRAAs often have more than one efferent branch, and so a level of renal parenchymal perfusion may have to be sacrificed if used in this situation. Additionally, unfavourable anatomy of the renal arterial origin or tortuosity may lead to difficulty with placing the device into a suitable position, as the size of the required guiding sheath/catheter can be large. It is also important to consider the potential diameter discrepancy between the two landing zones on either side of the TRAA, as they are often centred on renal arterial bifurcations [12].

### Follow-up

Patients were followed up clinically and/or with imaging. There was no standardised follow-up protocol; the interval and type of imaging follow-up was left to the individual operating interventional radiologist and specific patient co-morbidity in each case. When performed, follow-up imaging was either by ultrasound (colour flow and/or contrast-enhanced) or CT angiography. Imaging and/or clinical follow-up at or beyond 30 days was available for 13/18 interventions (72.2%) [mean 14.8 months; median 8 months; range 1–51 months]. With this data, a 30-day mortality analysis was also performed.

## Results

### Technical/clinical success and reintervention

**Fig. 6:**
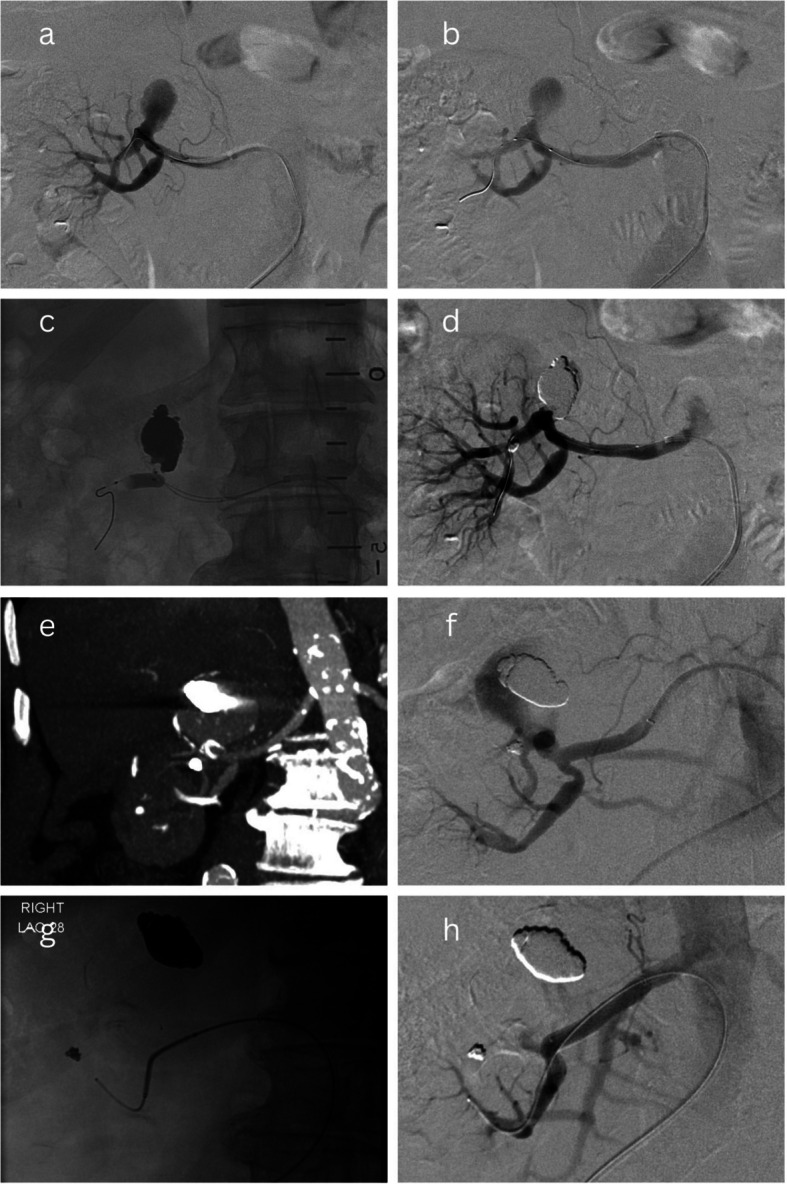
Reintervention case 3. A partially thrombosed 25 mm saccular Type 2 Rundback aneurysm (**a**) was treated endovascularly by manipulating a wire across the aneurysm into an efferent branch and positioning a balloon across the aneurysm’s origin (**b**). A separate microcatheter was placed in the aneurysm sac. The balloon was inflated and the sac was filled with Onyx until successful exclusion (**c**-**d**). CT imaging 8 years later incidentally demonstrated reperfusion and enlargement of the aneurysm (**e**). At reintervention, multiple attempts to cannulate the superior efferent branch were unsuccessful due to excessive tortuosity (**f**) and so a covered stent was placed from the main renal artery into the inferior segmental branch (**g**), successfully excluding the aneurysm but simultaneously sacrificing approximately 60% of renal parenchymal perfusion (**h**)

The technical success rate was 100% for the 15 TRAAs treated. The clinical success rate was 80% as reintervention was required in 3/15 cases (20%):

1. Presentation with acute right loin pain, bleeding, and haematuria four years after initial treatment of a saccular Type 2 Rundback TRAA, with CT demonstrating reperfusion and enlargement of the TRAA (from 35 to 56 mm). Initial intervention had been performed with balloon-assisted Onyx embolisation, with no sacrifice of renal perfusion. Successful reintervention was performed on an emergent basis, with placement of multiple endovascular occlusion systems and plugs into the TRAA sac and neck. Approximately 66% of renal perfusion had to be sacrificed (Fig. 2). Over time, the kidney became atrophic and non-functioning, but sCr remained within the normal range.
2. Partial reperfusion of a saccular Type 2 Rundback TRAA discovered on surveillance CT imaging during the first year after initial intervention. The initial intervention had been
  stent-graft placement across the proximal renal bifurcation (and the sac) into one of two efferent branches, at the cost of 10–30% renal perfusion, as vascular anatomy was not appropriate for stent-assisted coiling. Angiography demonstrated an inadequate proximal seal. The recurrent TRAA was successfully treated by packing the sac with coils and proximally extending the stent-graft, with no further sacrifice of renal perfusion (Fig. 5).
3. Reperfusion of a saccular Type 2 Rundback TRAA, incidentally discovered on abdominal imaging, almost 8 years after initial treatment with a balloon-assisted Onyx embolisation technique. Successful reintervention was performed by stent-graft placement into a separate segmental branch due to the inaccessibility of an efferent branch of the TRAA. As a result, approximately 60% of the superior polar arterial supply had to be sacrificed (Fig. 6).

No patients required conversion to nephrectomy and the 30-day mortality rate was 0% (for the 13/18 interventions where follow-up at or beyond 30 days was available).

### Renal parenchymal loss

Renal parenchymal perfusion loss estimated at completion angiography was < 10% for 10/15 TRAAs (66.7%), 10–30% for 2/15 TRAAs (13.3%), and 30–50% for 3/15 TRAAs (20%) at first intervention. At reintervention, 2/3 cases had 60–70% estimated angiographic renal parenchymal perfusion loss as the TRAAs had to be treated more aggressively. The other case of reintervention had < 10% estimated renal parenchymal perfusion loss. This has been summarised in Table 4.

**Table 4:** Post-treatment renal parenchymal perfusion loss, estimated at completion angiography

Renal parenchymal perfusion loss	
**At first intervention**	***N*** = **15**
0–10%	10 (66.7%)
10–30%	2 (13.3%)
30–50%	3 (20%)
**At reintervention**	***N*** = **3**
0–10%	1 (33.3%)
60–70%	2 (66.7%)

No patients required sacrifice of the entire renal arterial supply due to intraprocedural rupture.

### Renal function

Impact on renal function was assessed by statistically comparing pre- and post-procedural sCr for any significant difference. For the first intervention, the mean pre-procedural sCr was 78.7 µmol/L and early post-procedural sCr (taken at approximately 24 h post-procedure) was 94.5 µmol/L (normal range 62–98 µmol/L). Using a two-tailed, paired *t* test, the difference was found to be statistically insignificant (p > 0.05). No patients were readmitted with renal failure or subsequently developed chronic renal failure.

For the three repeat interventions, the mean pre-procedural sCr was 74 µmol/L and early post-procedural sCr was 98 µmol/L. A two-tailed, paired *t* test also found this to be statistically insignificant (p > 0.05).

### Complications

Of the 18 procedures performed (including reintervention), there was one case of non-target Onyx embolisation where a small piece of Onyx fragmented and embolised into an adjacent interlobar artery. This was non-occlusive with no significant associated loss of renal parenchymal perfusion, and so retrieval was not attempted. In addition, one patient developed a brachial artery access site pseudoaneurysm, which was successfully treated with thrombin injection. Another patient developed access site cellulitis (groin) shortly after their procedure. A third patient developed left common femoral vein deep venous thrombosis shortly after their procedure, where the adjacent left common femoral artery was accessed. As mentioned previously, there was one case of renal atrophy and loss of function. This patient’s sCr remained within the normal range, and there was no requirement for further post-procedural therapy. We also note that this occurred because of a proactive decision to sacrifice renal parenchymal perfusion during reintervention in the acute setting after the patient was admitted with loin pain and haematuria due to TRAA reperfusion 4 years after the initial intervention with balloon-assisted Onyx embolisation.

These complications have been summarised and graded in Table 5, according to The CIRSE Classification System [11]. The overall complication rate was 5/18 (27.8%).

**Table 5:** Summary and grading of complications according to The CIRSE Classification System [11]

Complications (5/18, 27.8%)	Grade
Non-target Onyx embolisation	1
Renal atrophy (sCr remained in normal range)	2
Brachial artery access site pseudoaneurysm	3
Access site cellulitis	3
Common femoral vein thrombosis	3

A summary of all treated cases has been provided in Table 6, detailing aneurysm characteristics, endovascular technique used, follow-up, and outcomes.

**Table 6:** Summary of TRAA characteristics, endovascular techniques and outcomes of all treated cases

TRAA	Rundback type	Size (mm)	Endovascular technique	Embolics used	Complications (grade)	Renal parenchymal perfusion loss (%)	Technical success (Y/N)	Follow-up (months)
1	2	12	SPEM	Onyx	0	0–10	Y	6
2	2	25	BAOE	Onyx	0	0–10	Y	40
-Reintervention		59	SG	-	0	60–70	Y	None/Unavailable
3	2	10	BAOE	Onyx	0	0–10	Y	1
4	2	10	BAOE	Onyx	Non-target Onyx embolisation (1)	0–10	Y	51
5	2	35	BAOE	Onyx	0	0–10	Y	6
-Reintervention		56	SPEM	Occlusion systems and plugs	Renal atrophy (2)	60–70	Y	9
6	2	34	SPEM	Coils and Onyx	0	30–50	Y	19
7	2	23	SAC	Coils	0	10–30	Y	None/Unavailable
8	2	22	BAOE	Onyx	0	30–50	Y	None/Unavailable
9	2	28	SAC	Coils	0	0–10	Y	18
10	2	19	SPEM	Occlusion systems, plugs and coils	0	0–10	Y	None/Unavailable
11	2	37	SG	-	0	10–30	Y	8
-Reintervention		37	SAC	Coils	Access site cellulitis (3)	0–10	Y	1
12	2	11	SAC	Coils	0	0–10	Y	5
13	2	5	SPEM	Coils	Common femoral vein thrombosis (3)	0–10	Y	None/Unavailable
14	1	40	PPMA	Plugs	0	30–50	Y	26
15	2	32	SAC	Coils	Brachial pseudoaneurysm (3)	0–10	Y	3

*Abbreviations*: *SPEM* sac packing with embolic material, *BAOE* balloon-assisted Onyx embolisation, *SG* stent-graft placement, *SAC* stent-assisted coiling, *PPMA* plug placement in proximal main artery

## Discussion

The literature demonstrates that EVT of TRAAs is safe and achieves high technical success [8, 13], which our study also supports. A limitation of our study is the absence of a comparator group. Open surgical repair (OR) has historically been the standard of care due to good reported outcomes for TRAAs. Large surgical studies, such as those by Henke et al. and Stanley et al., report perioperative mortality below 1% and durable aneurysm exclusion with long-term renal preservation [14, 15]. However, these benefits come at the expense of greater procedural morbidity, longer hospitalisation, and increased cardiopulmonary risk compared with minimally invasive options. In contrast, EVT achieves comparable technical success while reducing cardiopulmonary risk and hospital stay, though it carries a higher requirement for imaging surveillance and reintervention. This is evidenced by a recent large systematic review and meta-analysis by Choksi et al., which found that length of stay is significantly higher for OR in comparison to EVT (likely due to prolonged wound healing), while having comparable survival and morbidity outcomes with no significant difference in size and location of the TRAAs treated [16]. Interestingly, EVT did not demonstrate fewer complications, despite its less invasive nature, a finding corroborated by another large systematic review and meta-analysis [17]. This finding may be confounded by patient selection potentially being stricter for OR as it is more invasive and requires general anaesthesia, while EVT maintains the ability to treat a frailer patient cohort under local anaesthesia. This trade-off is increasingly relevant in modern practice where patient comorbidity and frailty may preclude open surgery. Practice at our centre aligns with a broader shift towards EVT.

The most frequent adverse event relating to EVT for TRAAs is renal ischaemia, demonstrated in Sheahan et al.’s recent qualitative systematic review of EVT of 454 TRAAs, which reported a rate of 11.5% [13]. Note, the reporting of renal ischaemia in this review was heterogenous, with most studies defining it at follow-up CT angiography, others at immediate post-treatment angiography, and others only reporting it if there was a functional correlate (e.g., impact on biochemical renal function). Nonetheless, this review emphasises that while it is a common adverse event, it is generally clinically well tolerated. This is a finding corroborated in our study, which demonstrates low rates of renal parenchymal loss and no associated adverse clinical manifestation. Note, renal function in this study was assessed using sCr alone, which is limited by it being an indirect measure. DMSA scintigraphy would have provided a more comprehensive assessment, particularly of differential and segmental renal function, but was not routinely employed due to practical reasons (minimising patient radiation dose, particularly when clinical concern for significant renal ischaemia was minimal). This limits the strength of our conclusions regarding the effect of EVT of TRAAs on renal function.

The mean TRAA sac size at initial intervention was 22.9 mm in this study, which is inconsistent with the suggested treatment threshold of > 30 mm by the SVS guidelines of 2020 [7], but consistent with the > 20 mm threshold suggested by the CIRSE Standard of Practice document of 2024 [8]. This is because most cases were treated prior to the publication of SVS’ guidelines, when the accepted treatment threshold was > 20 mm (based on a large retrospective surgical series by Henke et al. [14]). This finding was also observed in the meta-analysis by Choksi et al. reporting a mean sac size of 22.8 mm for 373 TRAAs treated endovascularly [16].

Regarding optimal endovascular technique, paramount considerations are aneurysm morphology and location in relation to segmental renal artery bifurcations. There is no published consensus regarding optimal endovascular technique. Generally, sac packing with embolic material is favoured for saccular aneurysms with narrow necks; wider necked saccular aneurysms can be more safely embolised with concurrent remodelling techniques with bare stents or temporary balloon inflation; and stent-graft placement is favoured for aneurysms of the main renal artery (Type 1 Rundback) with wider necks. Although there were no Type 3 Rundback TRAAs in this study, these are usually treated with liquid and/or coil embolic agents to occlude the aneurysm and its parent artery as the aneurysm lumen is often larger than the parent artery, and the resultant loss of renal parenchyma is usually not clinically significant due to the distal location of the TRAA [18]. However, the anatomy of TRAAs often does not clearly fit into one of these categories. In practice, the decision on which technique to use should be based on the specific anatomy of the aneurysm to be treated, as well as the experience and preference of the operator.

Notably, no flow diverter stents (FDS) were used in this study. Evidence for the use of such stents remains limited. Smaller case series have been published, such as Eldem et al.’s study of 2019, which included three patients treated with FDS, achieving 100% technical and clinical success at 12 months with no major complication [19]. Tipaldi et al.’s recent systematic review also compiled outcomes for a total of approximately 99 visceral aneurysms treated with FDS; this also suggested their potential safety and high efficacy, while maintaining branch vessel patency [20]. However, it should be noted that only 12 of these aneurysms were renal. Further, in both of these studies, FDS patency beyond 12 months for TRAAs is not certain. In-stent stenosis/occlusion was also highlighted as an issue in Tipaldi et al.’s systematic review, and the requirement for dual antiplatelet therapy is a limitation for FDS, particularly as TRAA patients can be young. Overall, the literature on TRAA treatment with FDS is limited and low-level, with most studies being single-centre case series. Confirmed rates of occlusion, procedural protocols, handling of complications, long-term patency, and reintervention are still uncertain, and larger multicentre studies are required to enrich the literature. It is possible that cases amenable to treatment with FDS are also amenable to stent-assisted coiling, which is our preferred technique, and so the role of FDS is also likely to relate to operator preference.

This study reports a reintervention rate for EVT of 3/15 (20%), with most of the earlier cases being treated with Onyx. Of these reinterventions, 2/3 (66%) were treated with Onyx alone, and we hypothesise that this may not be a durable technique. This could potentially be explained by Onyx not adhering to the endothelial wall of the sac (in contrast to other adhesive liquid embolics, e.g., cyanoacrylate), leaving greater potential for reperfusion and fragmentation of the liquid embolic post-procedure. This hypothesis is not confirmed and would require further research with direct comparative analysis and a larger sample size. Nonetheless, it has led to a shift towards stent-assisted coiling at our centre, with which we are yet to see any cases of reperfusion/reintervention. This technique has been illustrated in Fig. 3.

As mentioned, this study is limited by its retrospective and single-centre design and small sample size. This prevented a reliable direct comparison between different endovascular techniques. The heterogeneity of follow-up protocols represents an additional limitation, as variable surveillance protocols may under- or over-estimate long-term outcomes, complicating the assessment of durability and reintervention rates. Nonetheless, this study still provides important insights into the durability of EVT, with reinterventions being required up to 8 years after initial intervention. Despite its limitations, this study provides rare long-term follow-up data, while highlighting the need for multicentre studies to further define optimal techniques and surveillance protocols.

## Conclusion

EVT of TRAAs has a high rate of technical success with low complications, including low impact on renal perfusion and function. A higher rate of reintervention for Rundback Type 2 aneurysms treated with balloon-assisted Onyx embolisation was observed. Stent-assisted coiling is our preferred endovascular technique when this is anatomically feasible.

## Data Availability

Data can be acquired upon reasonable request to the corresponding author.
